# A novel class of oxynitrides stabilized by nitrogen dimer formation

**DOI:** 10.1038/s41598-018-32909-x

**Published:** 2018-09-27

**Authors:** Sangtae Kim, Hyo Jin Gwon, Sung Wook Paek, Seong Keun Kim, Ji-Won Choi, Jin-Sang Kim, Jung-Hae Choi, Chong-Yun Kang, Seung-Hyub Baek

**Affiliations:** 1 Center for Electronic Materials, Korea Institute of Science and Technology, Seoul, 02792 Republic of Korea; 2 Department of Materials Science and Engineering, Korea University, Seoul, 02841 Republic of Korea; 3 Materials R&D Center, Samsung SDI, Gyeonggi-do, 16678 Republic of Korea; 4 KU-KIST Graduate School of Converging Science and Technology, Korea University, Seoul, 02841 Republic of Korea; 5 Division of Nano & Information Technology, KIST School, Korea University of Science and Technology, Seoul, 02792 Republic of Korea

## Abstract

Despite the wide applicability of oxynitrides from photocatalysis to refractory coatings, our understanding of the materials has been limited in terms of their thermodynamics. The configurational entropy via randomly mixed O/N or via cation vacancies are known to stabilize oxynitrides, despite the positive formation enthalpies. Here, using tin oxynitrides as a model system, we show by *ab initio* computations that oxynitrides in seemingly charge-unbalanced composition stabilize by forming pernitrides among metal-(O,N)_6_ octahedra. The nitrogen pernitride dimer, =(N-N)=, results in the effective charge of −4, facilitating the formation of nitrogen-rich oxynitrides. We report that the dimer forms only in structures with corner-sharing octahedra, since the N-N bond formation requires sufficient rotational degrees of freedom among the octahedra. X-ray photoemission spectra of the synthesized tin oxynitride films reveal two distinct nitrogen bonding environments, confirming the computation results. This work opens the search space for a novel kind of oxynitrides stabilized by N dimer formation, with specific structural selection rules.

## Introduction

Doping or mixing different species of anions in ceramics’ lattice may extend the known horizon of materials properties. Oxynitrides (or equivalently oxide nitrides) are one example where oxygen and nitrogen compose the anion sublattice^1^. The discovered oxynitrides have proven to be useful engineering materials in various applications including photocatalysts^2^, passivation layers^3^, refractory materials^4^ or LED phosphors^5^. Compared to cation mixed ceramics, however, understanding on oxynitrides has been limited in terms of their crystal structures, compositions and elements comprising oxynitrides. The number of synthesized crystalline oxynitrides falls far below those of oxides or nitrides, with some well-known oxynitrides including perovskite-based oxynitrides^6,7^, SiO_*x*_N_*y*_^5^, AlO_*x*_N_*y*_^8^, GaO_*x*_N_*y*_^9^, TaO_*x*_N_*y*_^10^, or more complex ones with several cations such as SiAl_*z*_O_*x*_N_*y*_^3^. A significant portion of vacancies and the following configurational entropy have been argued to stabilize the structure despite the positive enthalpy of formation^9,11,12^.

Recently discovered crystalline tin oxynitrides, however, calls for further understanding on the thermodynamics of oxynitride formation^13^. Sputtering rutile SnO_2_ under ammonia/nitrogen environment results in nitrogen-rich tin oxynitrides in fluorite-type (Spacegroup: *Pa*$\bar{3}$) structure. The compound forms only in this specific phase, suggesting that the thermodynamics of phase selection in oxynitrides may differ significantly from those in its oxide counterparts; fluorite SnO_2_ is a high-pressure phase that forms at above 10 GPa. In addition, the nitrogen-rich composition calls for questions in the charge balancing problem. Since tin does not form 5+ oxidation state, each nitrogen incorporation into fluorite SnO_2_ requires oxygen vacancy formation to meet the charge neutrality:

$$3{{\rm{O}}}_{{\rm{O}}}^{x}+{N}_{2}=2{N^{\prime} }_{O}+{{\rm{V}}}_{{\rm{O}}}^{\cdot \cdot }+\frac{3}{2}{O}_{2}$$

To form nitrogen-rich tin oxynitrides according to this scheme, therefore, the material must have unreasonable vacancy concentration of over 0.5 vacancies per Sn atom. In similar experiments, attempts to substitute oxygen with nitrogen in perovskite BaTiO_3_ results in nitrogen content only up to BaTiO_2.8+δ_N_0.1_^14^. For elements capable of penta-valence cations, however, significant portion of nitrogen gets incorporated; nitriding BaTaO_3_ results in BaTaO_2_N, owing to the formation of Ta^5+^ cation^15^. The formation of nitrogen-rich tin oxynitrides, therefore, suggests that additional stabilization mechanism to configurational entropy may exist for the ceramics.

**Figure 1:**
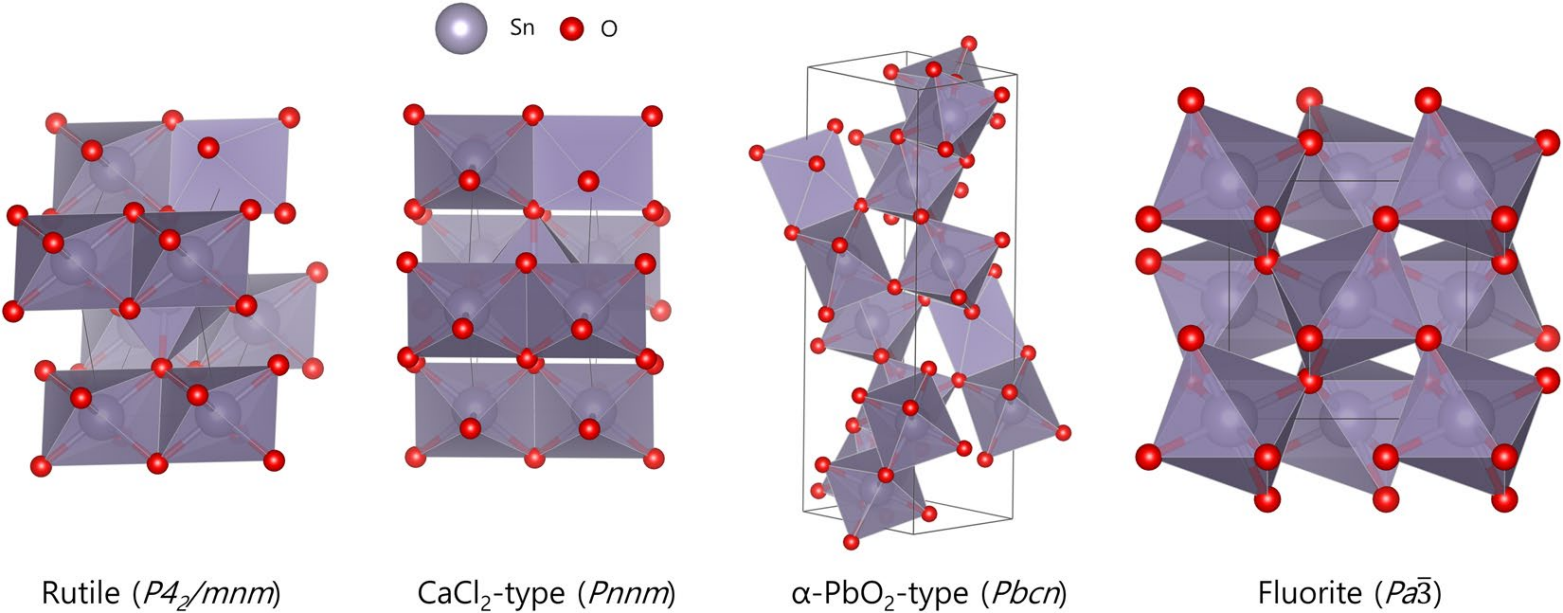
The four polymorphs of SnO_2_. The highlighted octahedra show the polyhedral connectivity, with corner-sharing octahedra in fluorite.

Here, we use density functional theory (DFT) computations to study the energetics of phase selection among tin oxynitride polymorph phases. For 7 different O-N substitution ratios, the formation energies are compared in four well-known SnO_2_ polymorph structures, namely the rutile (*P*4_2_*/mnm*), CaCl_2_-type (*Pnnm*), α-PbO_2_ type (*Pbcn*) and the fluorite-type (*Pa*$\bar{3}$) structures (Fig. 1). To study whether specific O-N ordering affects the thermodynamics of oxynitrides, we study 30 symmetrically distinct O-N configurations for each structure and composition. We find that specific O-N configuration leads to the nitrogen-nitrogen covalent bond formation and significantly stabilizes the fluorite phase as opposed to the other phases unaffected by O-N configurations. Experimental characterization efforts reveal nitrogen-nitrogen bonding in the synthesized thin films, confirming the results of the computation.

## Results

**Figure 2:**
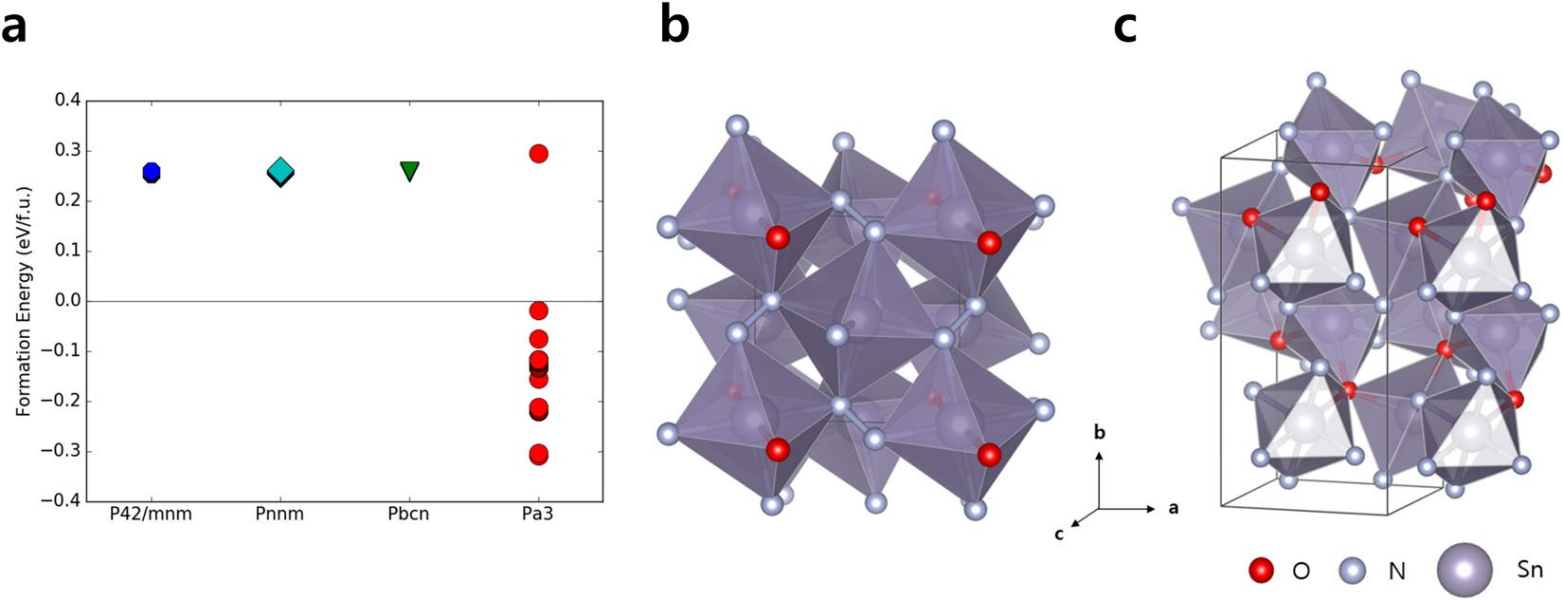
(**a**) The computed formation energies of SnO_0.5_N_1.5_ at different O-N orderings. (**b**,**c**) The computed structures with (**b**) the lowest formation energy and (**c**) the highest formation energy.

Figure 2a illustrates the formation energies of tin oxynitride SnO_0.5_N_1.5_ at different O-N orderings. The formation energies of cubic fluorite phase (*Pa*$\bar{3}$) show a large spread between −0.31 eV/f.u. and 0.29 eV/f.u. among different O-N configurations, while those of the other crystal structures remain nearly fixed at 0.26 eV/f.u. The positive formation energies observed for these structures indicate that the oxynitride formation is not favored over their constituent elements in elemental forms (Sn metal, O_2_ and N_2_ gas). The negative formation energies of some fluorite SnO_0.5_N_1.5_ show that specific O-N orderings stabilize fluorite structure more than others. This general trend persists in most compositions considered, except for the oxygen-rich SnO_1.67_N_0.33_ composition (Supplementary Fig. S1).

The amount of formation energy variation induced by O-N ordering clearly exceeds the expected ordering-dependent energy scale. For example in melilite type Y_2_Si_3_O_3_N_4_ oxynitride, specific O-N ordering has been verified by both experimental and computational work^11,16^. The lowest energy O-N ordering that is also consistent with the experimentally observed case differs only by 74 meV per two anions from the highest energy ordering^11^. This is much less than the 603 meV observed among O-N orderings in fluorite SnO_0.5_N_1.5_ (*Pa*$\bar{3}$) phase. The other structures do not get affected by the different O-N orderings, with the energies between ordering varied by only 8 meV. The large energy difference in fluorite also exceeds the formation energy difference of 100 meV/f.u. between the ground state (rutile) and high-pressure phase (fluorite) SnO_2_ (Supplementary Fig. S2). This large enthalpy difference, on the order of bond breaking, signals that specific O-N ordering may lead to additional covalent bond formation.

Figure 2b,c illustrate the computed structures for the highest- (left) and the lowest-energy (right) O-N orderings in fluorite SnO_0.5_N_1.5_. In the lowest-energy configuration (Fig. 2b), we notice nitrogen atoms among Sn(O,N)_6_ octahedra bond to each other, forming a nitrogen dimer. The observed N-N bond length of 1.48 Å corresponds to the reported value of nitrogen single bond. All 6 nitrogen atoms in the unit cell participate in the pernitride formation. The oxygen atoms are arranged to form linear O-Sn-O bonds, with the average Sn-O bond lengths of 2.10 Å. This is close to the Sn-O bond lengths (2.09 Å) observed in rutile SnO_2_, when computed with the identical pseudopotentials. The average Sn-N bond length is 2.24 Å, close to the Sn-N bond lengths (2.20 Å) in octahedrally coordinated parts of Sn_3_N_4_. In the highest-energy configuration shown in Fig. 2c, on the other hand, we do not observe any nitrogen-nitrogen bonding. The nitrogen atoms in this configuration break Pauling’s second rule, since the nitrogen anions (N^3−^) coordinated by three tetravalent cations do not meet the local electroneutrality requirement^17^. The average bond lengths of Sn-O and Sn-N are 2.16 Å and 2.18 Å, respectively, challenging the natural bond lengths for Sn-O and Sn-N. The variance in bond lengths are also significant; the maximum and minimum Sn-N bond lengths are 2.12 Å and 2.23 Å, indicating that significant local strains exist within the unit cell.

The lowest-energy configuration yields reduced unit cell volume (34.4 Å^3^/f.u.) compared to that (37.35 Å^3^/f.u.) of the high-energy configuration (Supporting Table S1). The 8% volume difference shows that nitrogen dimer formation leads to the more tightly packed material in the same composition. The density for the lowest-energy configuration is 7.23 g/cm^3^, notably higher than that of rutile SnO_2_ (6.61 g/cm^3^) or that of the highest-energy configuration (6.66 g/cm^3^). The increased density of tin oxynitrides despite the inclusion of larger-sized nitrogen atoms is in good agreement with the experimental observations that the synthesized tin oxynitride thin films possess higher density (7.49 g/cm^3^) than the rutile SnO_2_ (6.99 g/cm^3^)^13^.

The pernitride formation is observed consistently in fluorite SnO_2−*x*_N_*x*_ in all seven compositions we considered. At each composition, the lowest-energy configuration in fluorite phase always involves the nitrogen dimer formation, even when fluorite is not the ground state at SnO_1.67_N_0.33_ composition. While such anion-anion bonding has rarely been observed in oxide ceramics^18^, it has been observed in some classes of transition metal nitrides. For instance, PtN_2_ has been reported to form N-N bonding among PtN_6_ octahedra, with the nitrogen dimers exhibiting valences of −4 (Supplementary Fig. S3)^19,20^. Since the nitrogen atoms participate in forming a covalent single bond with each other, the effective valences of the pernitrides [=N-N=] are −4, with each nitrogen being effectively −2 valence. This unique feature to nitrogen-containing compounds effectively solves the charge neutrality problem, eliminating the need for excessive vacancy formation mentioned in the Introduction.

**Figure 3:**
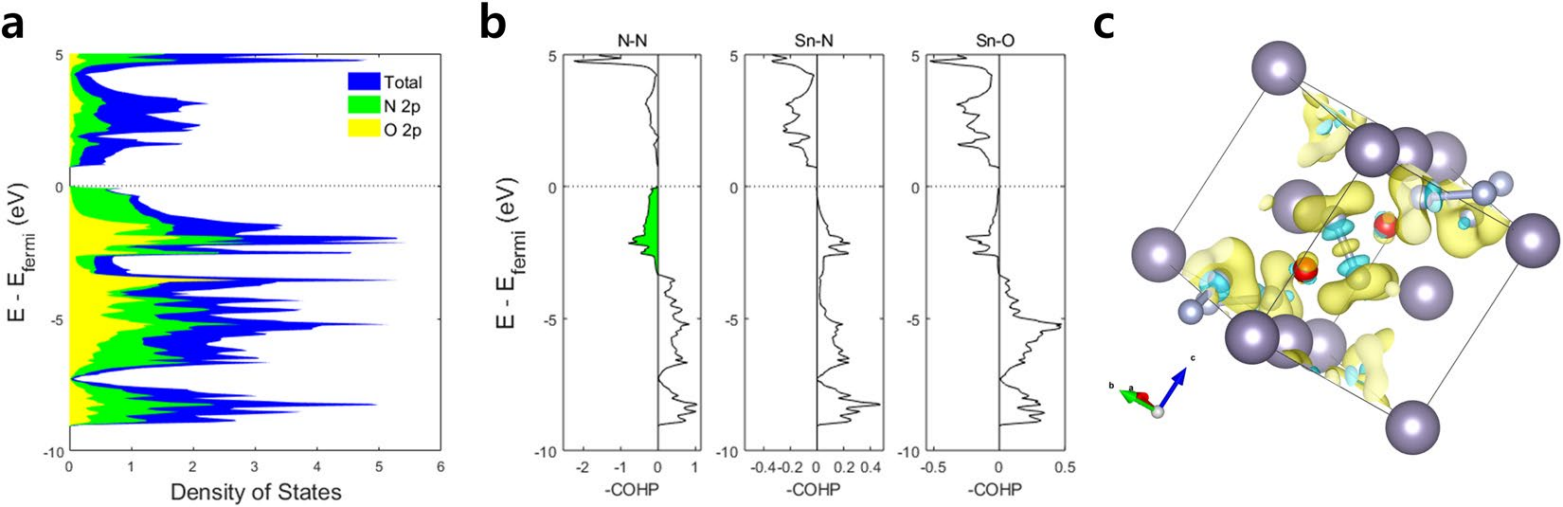
The electronic structure of the lowest-energy SnO_0.5_N_1.5_ configuration. (**a**) The atomic orbital projected density of states for the material, with the total density of states shown in blue. (**b**) The crystal orbital Hamilton population for N-N, Sn-N and Sn-O interactions. (**c**) The charge density difference plot within the unit cell. The isosurfaces show 0.015 e^−^ per Bohr radius^3^, with yellow bubbles indicating gained electron density while the blue bubbles reduced electron density.

The density of states (DOS) and crystal orbital Hamilton population^21,22^ (COHP) shown in Fig. 3 reveal the electronic structure of the lowest-energy SnO_0.5_N_1.5_ configuration and the nature of its N-N bonding. The atomic orbital-projected DOS in Fig. 3a reveal that N 2p orbitals are responsible for the reduced band gap of the oxynitride compared to the oxide counterpart. The COHP diagrams in Fig. 3b show bonding orbitals to the right of the neutral axis and antibonding orbitals to the left. We notice filled antibonding orbitals among N-N interaction, with the antibonding states reaching down to −3.35 eV from the Fermi level. The qualitative molecular orbital diagrams in Supplementary Fig. S4 show that a nitrogen dimer fills the antibonding states, only when the dimer accepts additional electrons from its neutral state. Similar, yet more comprehensive diagrams have been reported in the literature^20^. The completely filled antibonding band observed in Fig. 3a corresponds well to the [=N-N=]^4−^ states, with 1π* orbitals filled up with four additional electrons.

Interestingly, the Sn-N interaction in SnN_6_ octahedra shows only the bonding states, with unfilled antibonding states above the Fermi level. This contrasts the Sn-N interaction in Sn_3_N_4_ where we clearly observe low-density yet filled antibonding states (Supplementary Fig. S5). Sn-O interaction, on the other hand, shows consistently filled antibonding states in both SnO_0.5_N_1.5_ and rutile SnO_2_, indicating that Sn-O interaction does not get affected by N-N dimer formation as much as the Sn-N interaction. In the charge density difference plot (Fig. 3c), the electron density between the two neighboring N atoms increases at the center and decreases between the center point and the atoms. This indicates that the formation of N-N bond is covalent, with electron cloud shifting towards the center compared to their atomic states. We also notice that the electron density isosurface around each N atom has three large blobs towards the Sn atoms that the N bonds to. This shows that Sn’s electron cloud shifts towards N from Sn, reflecting partially ionic character of the Sn-N bonds. At the same electron density isosurface, however, we do not observe as much electron cloud shift towards oxygen, although the electronegativity difference is greater between Sn and O than that between Sn and N. This suggests that the formation of N-N dimer adds some ionic character of the Sn-N interaction.

**Figure 4:**
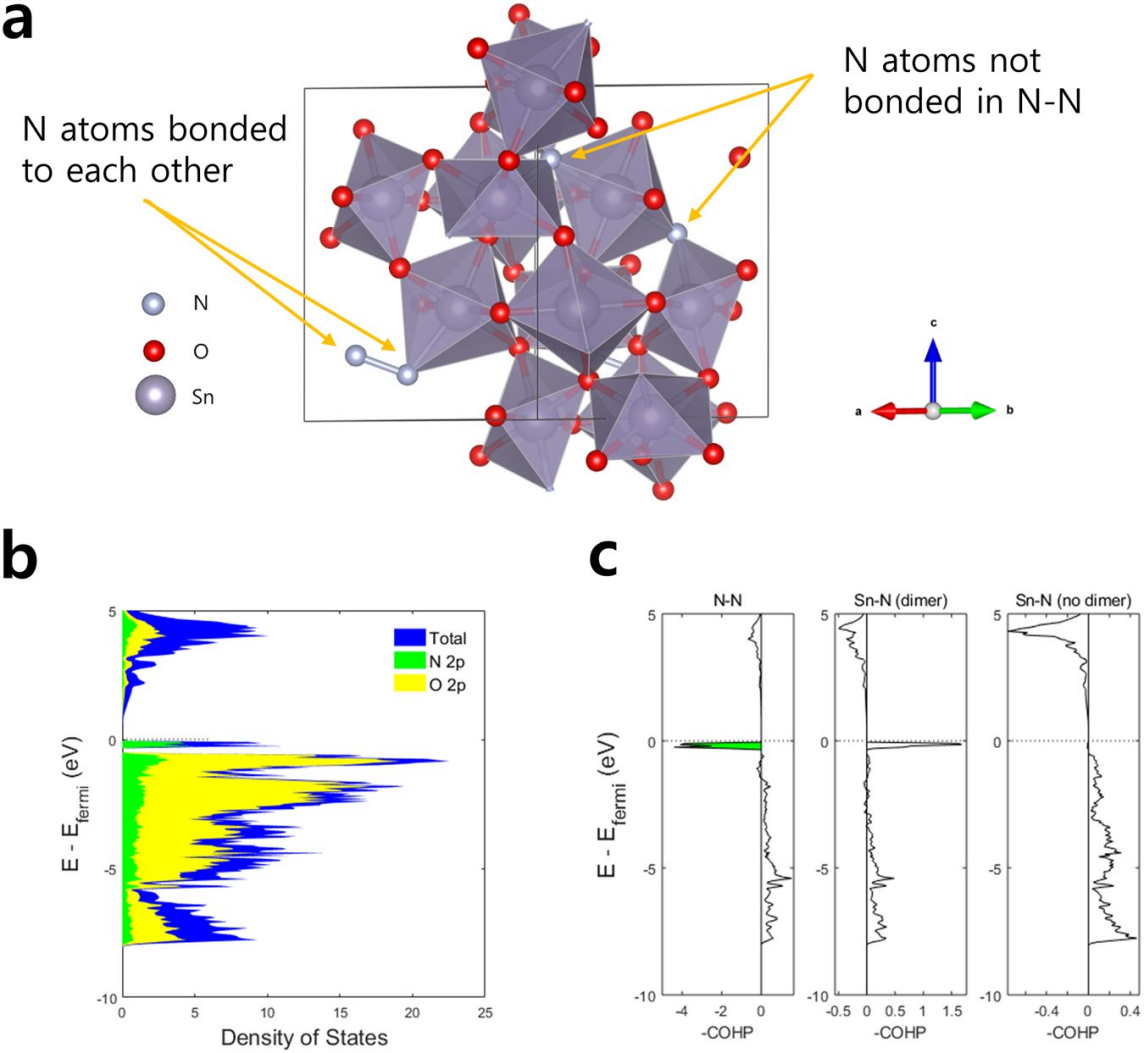
(**a**) The lowest-energy configuration for SnO_1.5_N_0.5_ composition. We notice two N atoms that are bonded to each other and two other N that are not. (**b**) The atomic orbital projected DOS for the material. (**c**) The COHP of the N-N and Sn-N interactions. Sn-N interactions are shown for N atoms that participate in dimer formation and for those that do not. The filled antibonding orbitals are colored in green.

Performing similar analyses on the lowest-energy SnO_1.67_N_0.33_ further confirm that N-N bond formation affects the Sn-N interaction. The unit cell for the lowest-energy SnO_1.67_N_0.33_ configuration contains 4 nitrogen atoms, with two forming N-N bonds while the other two not participating in N-N bond formation (Fig. 4a). The cell thus provides the model system to examine the difference in Sn-N interaction via COHP between the dimer-forming N atoms and regular N atoms that do not participate in pernitride formation. Figure 4b shows that the DOS has strongly localized states near the band gap, contributed largely by the N 2p orbitals. The COHP in Fig. 4c shows that the dimered N-N interaction results in strongly localized antibonding states just below the band gap. The Sn-N interaction of the dimered N atom shows strongly localized bonding states and no antibonding orbitals occupied, similar to those observed in SnO_0.5_N_1.5_. The Sn-N interaction of the non-dimered N in the same unit cell, however, shows COHP similar to that of Sn_3_N_4_, with glimpse of antibonding states near the band gap. This contrast clearly shows that N-N bond formation changes the nature of the Sn-N interaction, affecting the overall electronic structure and ionic character to the material.

**Figure 5:**
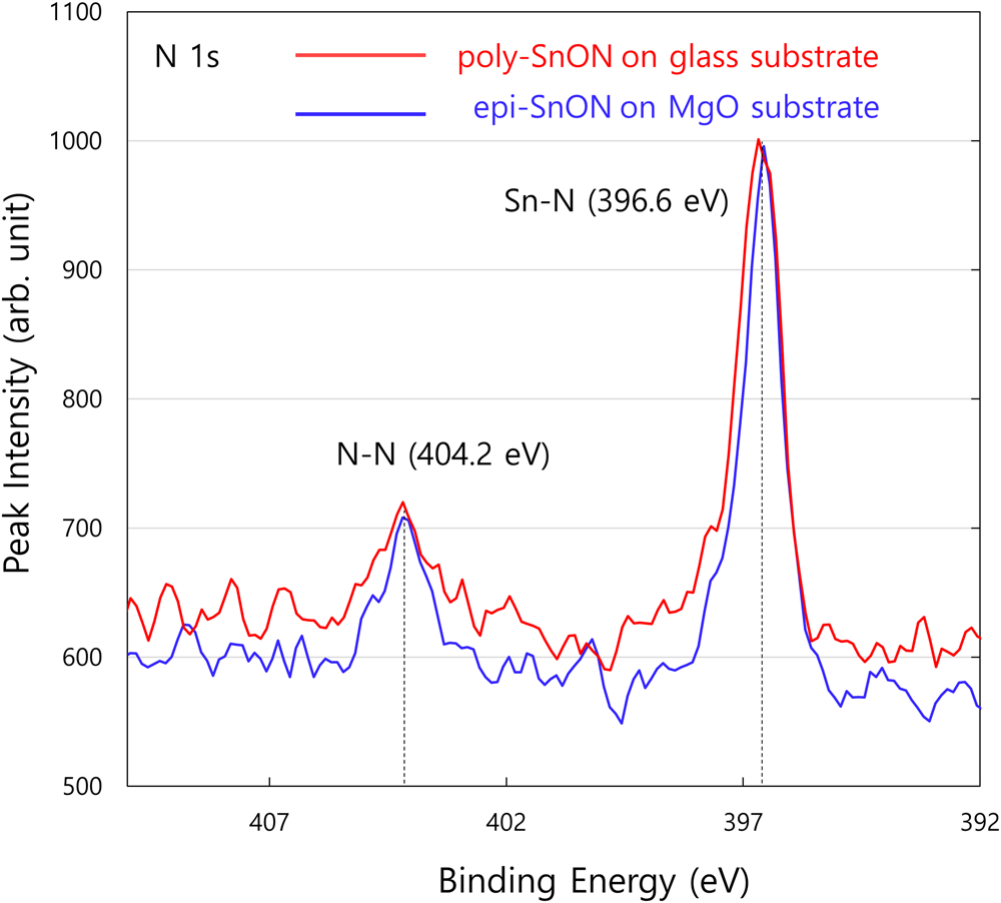
X-ray photoelectron spectra (XPS) of the synthesized tin oxynitride thin films reveals two distinct nitrogen bonding environment.

The existence of N-N bonding is confirmed experimentally by observing the X-ray photoemission spectra (XPS). Figure 5 plots the XPS obtained from the synthesized tin oxynitride thin films with the reported composition of SnO_0.39_N_1.26_^13^. The XPS spectra reveal two distinct bonding environment for nitrogen, one corresponding to Sn-N bonds at 396.6 eV and the other to N-N bonds at 404.2 eV. The two bonding environments consistently occur in both polycrystalline films grown on glass substrates and epitaxial films grown on MgO substrates. The grown films are reported to be in fluorite phase via X-ray diffraction, without any sign of secondary phases^13^. These results, in accordance with the computation results, confirm the existence of nitrogen dimers.

**Figure 6:**
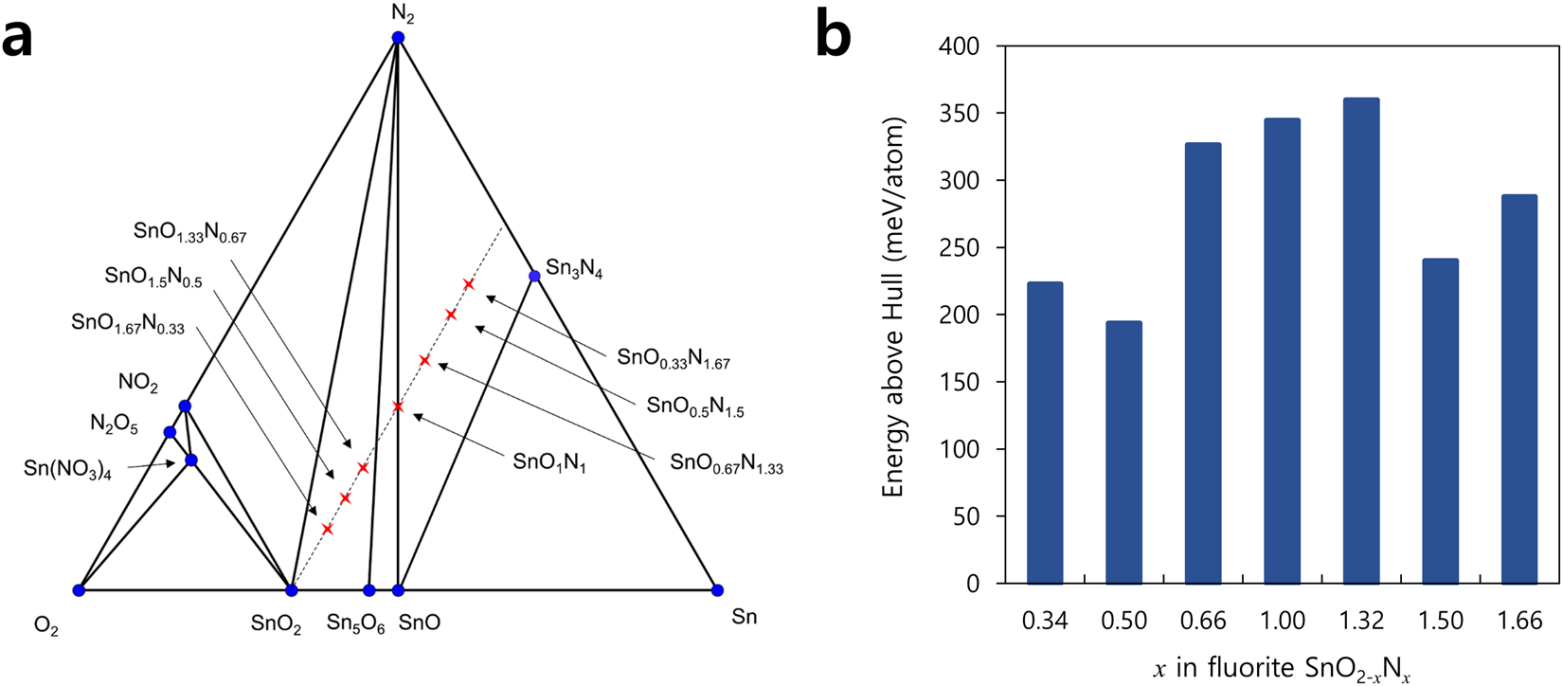
(**a**) The constructed phase diagrams of Sn-N-O chemical system. The blue dots indicate stable phases at 0 K and the metastable computed entries as red crosses. (**b**) The energy above hull for fluorite SnO_2−*x*_N_*x*_ according to the composition.

While the pernitride formation stabilize tin oxynitrides in fluorite structure, the 0 K phase diagram analysis shows that the configuration is metastable (Fig. 6a). The constructed phase diagram shows that there exists no stable tin oxynitride at 0 K, except for one nitrate phase (Sn(NO_3_)_4_). This instability owes in large part to the exceptional stability of N_2_ gas and Sn_3_N_4_, which are included in the decomposition products of the metastable oxynitrides. The energy hull construction in Fig. 6b reveals that the computed oxynitrides are metastable by at least 193 meV/atom for all composition, with the computed SnO_0.5_N_1.5_ structure metastable by 240 meV/atom. At this specific composition, the energy above hull for the other three structures reach up to ~805 meV/atom, showing that the nitrogen dimer stabilizes the fluorite structure by a significant amount.

## Discussion

Interestingly in our computation, the nitrogen-nitrogen bonding only occurs in fluorite tin oxynitrides; no O-N configuration in the other three polymorph structures results in the dimer formation. Considering that fluorite SnO_2_ is not the ground state, we question whether specific structural features favor the formation of pernitrides. Comparing the original fluorite structure with the DFT relaxed structure in Fig. 2b, we notice that the pernitride formation involves a significant amount of octahedral rotation (Supplementary Fig. S6). For some specific O-N ordering, the corner-sharing octahedra rotate collectively to form nitrogen dimers for all nitrogen atoms. It comes naturally from this observation that rigid structures that constrain polyhedra’s rotational degrees of freedom have difficulty nucleating the nitrogen dimer-stabilized oxynitrides. Among the four SnO_2_ polymorphs considered in this study, we notice that fluorite structure consists of only corner-sharing SnO_6_ octahedra, with each anion corner shared between three octahedra. In the other three structures, most octahedra share two edges among each other. The spinel Sn_3_N_4_ phase also consists of edge-sharing SnN_6_ octahedra and SnN_4_ tetrahedra^23^. The polyhedral network connected by edge-sharing polyhedra limits the rotational degree of freedom, possibly suppressing the formation of nitrogen dimers.

The nitrogen dimer stabilization does not yield fluorite as the ground state in oxygen-rich SnO_1.67_N_0.33_ composition, with the rutile phase being the ground state (Supplementary Fig. S1). The energetics at this composition remains more or less similar to SnO_2_, with the stability order of rutile, CaCl_2_-type, α-PbO_2_-type and fluorite-type structures. The lowest-energy O-N ordering in fluorite is 42 meV/f.u. higher in energy compared to the rutile phase, and involves two N atoms forming a dimer. The other two N atoms remain bonded only to three Sn atoms like the oxygen atoms. The presence of nitrogen atoms not participating in dimer may be caused by the incomplete sampling of the O-N ordering; 30 distinct configurations ranked by electrostatic summation may not provide the configuration with nitrogen atoms in neighboring pairs. However, we do observe that the stability of fluorite tin oxynitrides against the rutile phase linearly increases with the nitrogen substitution content (Supplementary Fig. S7). The more nitrogen gets substituted into SnO_2_, the more stable the fluorite structure becomes compared to the other polymorphs. These suggest that nitrogen dimer-stabilized oxynitrides favor nitrogen-rich composition, as opposed to the oxygen-rich oxynitrides stabilized via vacancies and their configurational entropy contribution.

Regarding the oxynitrides’ stability, the metasability of 200 meV/atom is considered large. However, Sun *et al*. recently showed that the thermodynamic scale of nitrides’ metastability reaches 192 meV/atom at 90^th^ percentile^24^. The large metastability of the computed oxynitride phases along with the reported synthesis of tin oxynitrides, therefore, suggest that the energetic barrier to decompose the oxynitrides may be large. This is likely due to the extra pernitride bond formation. The analyses in this work suggests that there may be a group of novel oxynitrides stabilized by nitrogen dimer formation yet to be discovered.

## Conclusion

In summary, we show by density functional theory computations that nitrogen-rich tin oxynitrides stabilize in fluorite phase by forming nitrogen dimers as opposed to the other polymorph structures of SnO_2_. The electronic structure confirms the covalent N-N bonding, and X-ray photoelectron spectra of synthesized tin oxynitride films reveal two distinct nitrogen bonding environment, consistent with the computation results. The formation of pernitrides requires sufficient nitrogen concentration in the anion sublattice and specific O-N configuration with nitrogen in neighboring pairs. For sufficient rotational degrees of freedom, structures with corner-sharing polyhedra network are favored over the edge-sharing or face-sharing polyhedra. This specific structural feature stabilizes the tin oxynitrides in the high-pressure polymorph form under ambient conditions. The mechanisms revealed in this work opens up the avenues for the discovery of other nitrogen-rich oxynitrides, particularly in metastable phases in their oxide counterpart.

## Methods

### Computation Details

Vienna *ab initio* simulations package was used to perform the density functional theory (DFT) calculations^25^. Generalized-gradient approximation (GGA) was used to treat the exchange-correlation functional and Perdew-Burke-Erzenhof functional with projector-augmented wave method was used^26,27^. All structural elements including cell parameters and ionic positions were relaxed with the energy cutoff of 520 eV. The K-point density of at least 1000/atom was used, and all calculations were spin-polarized. No Hubbard-like U correction was employed, and symmetry in the wavefunction was explicitly broken during calculation.

To study the thermodynamics of oxynitride formation, formerly known polymorphs of tin oxides were used as base structures, including rutile (*P4*_2_*/mnm*), CaCl_2_-type (*Pnnm*), α-PbO_2_ type (*Pbcn*) and cubic fluorite-type (*Pa*$\bar{3}$) (Fig. 1). For each structure, oxygen sites were partially substituted with nitrogen based on SnO_2−*x*_N_*x*_ formula, with substitution percentage of 17% (*x* = 0.33), 25% (*x* = 0.5), 33% (*x* = 0.67), 50% (*x* = 1), 67% (*x* = 1.33), 75% (*x* = 1.5) and 83% (*x* = 1.67). The O-N ordered structures at these pre-defined O-N ratios were generated using derivative structure enumeration library implemented by Hart *et al*.^28^. All symmetrically unique O-N orderings within 12 SnO_2−*x*_N_*x*_ (0.33 ≤ *x* ≤ 1.67) formula unit supercells were considered, and 30 structures with minimum Ewald summation were selected and calculated using density functional theory^29^. The oxidation states of Sn^4+^, O^2−^ and N^3−^ were assumed in the Ewald summation.

The formation energies of computed structures were evaluated according to the scheme by MaterialsProject^30^. The gas phase energies required for formation energy calculations were corrected according to the previous works by Wang *et al*.^31^. The decomposition products were assessed via phase diagram construction, implemented in the MaterialsProject API^32^. The COHP was obtained using Lobster^21,22,33,34^. The charge density difference plot was generated using the atomic states as the reference. The computed structures were visualized with VESTA^35^.

## Experimental Details

The tin oxynitride films are synthesized as reported in our previous publication^13^. XPS measurements are obtained for thin films grown at 75 W on glass and MgO substrates using PHI 5000 VersaProbe(Ulvac-PHI). The surface few layers of the films were etched before XPS measurement, to minimize the effect of surface oxidation and adsorbents. The background pressure was 6.7 × 10^−8^ Pa, and the XPS peaks were calibrated using C1s peak at 284.6 eV. The X-ray spot size was 100 μm × 100 μm.

## Electronic supplementary material

Supplemenatry Information
