# Supplementary material for: A novel class of oxynitrides stabilized by nitrogen dimer formation
Source: Sci Rep. 2018 Sep 27;8:14471. doi: 10.1038/s41598-018-32909-x (PMC6160415; doi:10.1038/s41598-018-32909-x)
Supplement: Supplementary file 1 — Supplemenatry Information [file 41598_2018_32909_MOESM1_ESM.docx]

**A novel class of oxynitrides stabilized by nitrogen dimer formation**

Sangtae Kim^1*^_,_ Hyo Jin Gwon^1,2^, Sung Wook Paek^3^, Seong Keun Kim^1^, Ji-Won Choi^1^, Jin-Sang Kim^1^, Jung-Hae Choi^1^, Chong-Yun Kang^4,1*^, Seung-Hyub Baek^1,5*^

1. Center for Electronic Materials, Korea Institute of Science and Technology, Seoul 02792, Republic of Korea
2. Department of Materials Science and Engineering, Korea University, Seoul 02841, Republic of Korea
3. Materials R&D Center, Samsung SDI, Gyeonggi-do 16678, Republic of Korea
4. KU-KIST Graduate School of Converging Science and Technology, Korea University, Seoul 02841, Republic of Korea
5. Division of Nano & Information Technology, KIST School, Korea University of Science and Technology, Seoul 02792, Republic of Korea.

* Correspondence should be addressed to S.K. ([stkim@kist.re.kr](mailto:stkim@kist.re.kr), +82-2-958-6623), C.-Y.K. ([cykang@kist.re.kr](mailto:cykang@kist.re.kr), +82-2-958-6722) and S.-H.B. (email: [shbaek77@kist.re.kr](mailto:shbaek77@kist.re.kr), +82-2-958-5382)

Keywords: oxynitride, nitrogen dimer, O-N ordering, *ab initio* thermodynamics, density functional theory

**Table of Contents**

1. **Figure S1.** The computed formation energies of SnO*_x_*N*_y_* at six different O-N substitution ratio. Fluorite phase is the ground state at all composition but the oxygen-rich SnO_1.67_N_0.33_.
2. **Figure S2.** The computed formation energies of the four SnO_2_ polymorphs. The rutile phase is the ground state, and the high-pressure fluorite phase is metastable by 100 meV/f.u..
3. **Figure S3.** The reported PtN_2_ pernitride structure (left) and the predicted SnO_0.5_N_1.5_ structure (right). The two structures exhibit similar nitrogen dimer formation among the octahedra.
4. **Figure S4.** The molecular orbital diagrams for the nitrogen dimer. The neutral N_2_ dimer does not fill any antibonding states from 2p orbitals. When additional electrons are involved, states from 1𝞹* orbitals get filled in.
5. **Figure S5.** The COHP of Sn-N interaction from the SnN_6_ octahedra in Sn_3_N_4_ and Sn-O from SnO_2_. Both interactions involve filled antibonding states that are colored in green.
6. **Figure S6.** The configuration before atomistic relaxation (left) and after relaxation (right) by density functional theory energy minimization process. The relaxation process reveals collective polyhedral rotation that forms N-N bonds.
7. **Figure S7**. The difference in formation energy between fluorite and rutile phase SnO_2-_*_x_*N*_x_* with respect to the nitrogen substitution ratio *x*.
8. **Table S1.** Lattice information for the lowest- and highest-energy SnO_0.5_N_1.5_ configurations


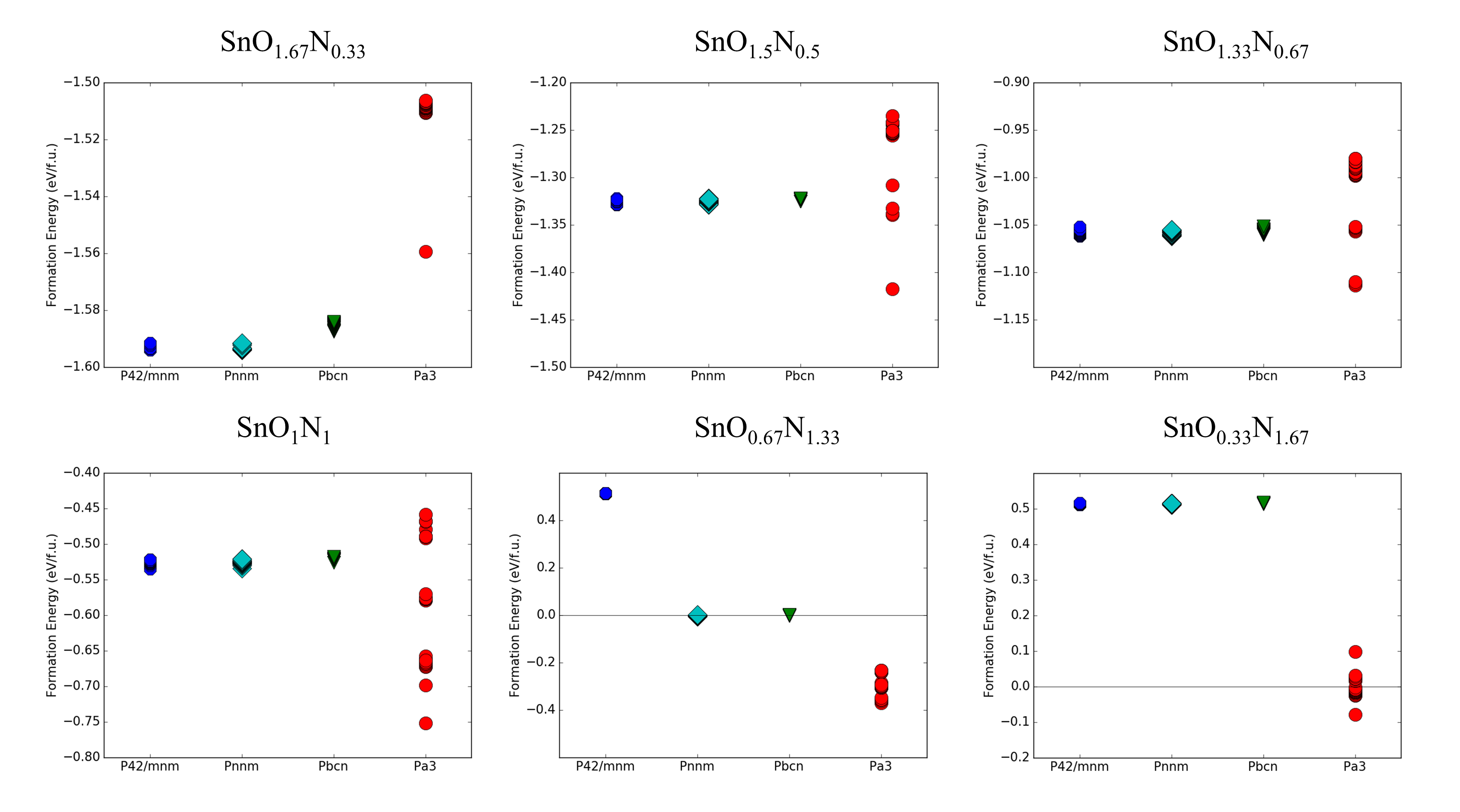


**Figure S1.** The computed formation energies of SnO*_x_*N*_y_* at six different O-N substitution ratio. Fluorite phase is the ground state at all composition but the oxygen-rich SnO_1.67_N_0.33_.


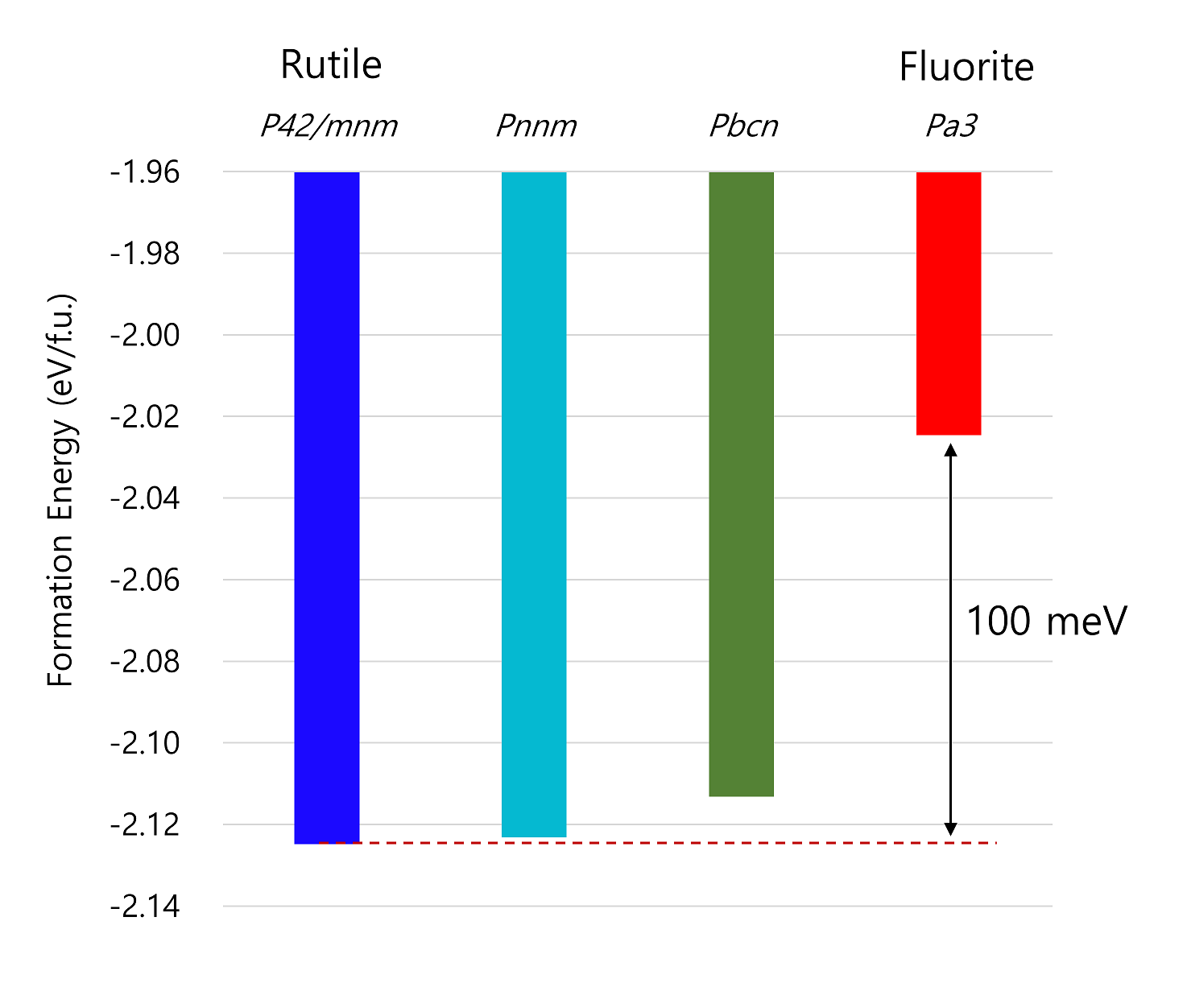


**Figure S2.** The computed formation energies of the four SnO_2_ polymorphs. The rutile phase is the ground state, and the high-pressure fluorite phase is metastable by 100 meV/f.u..


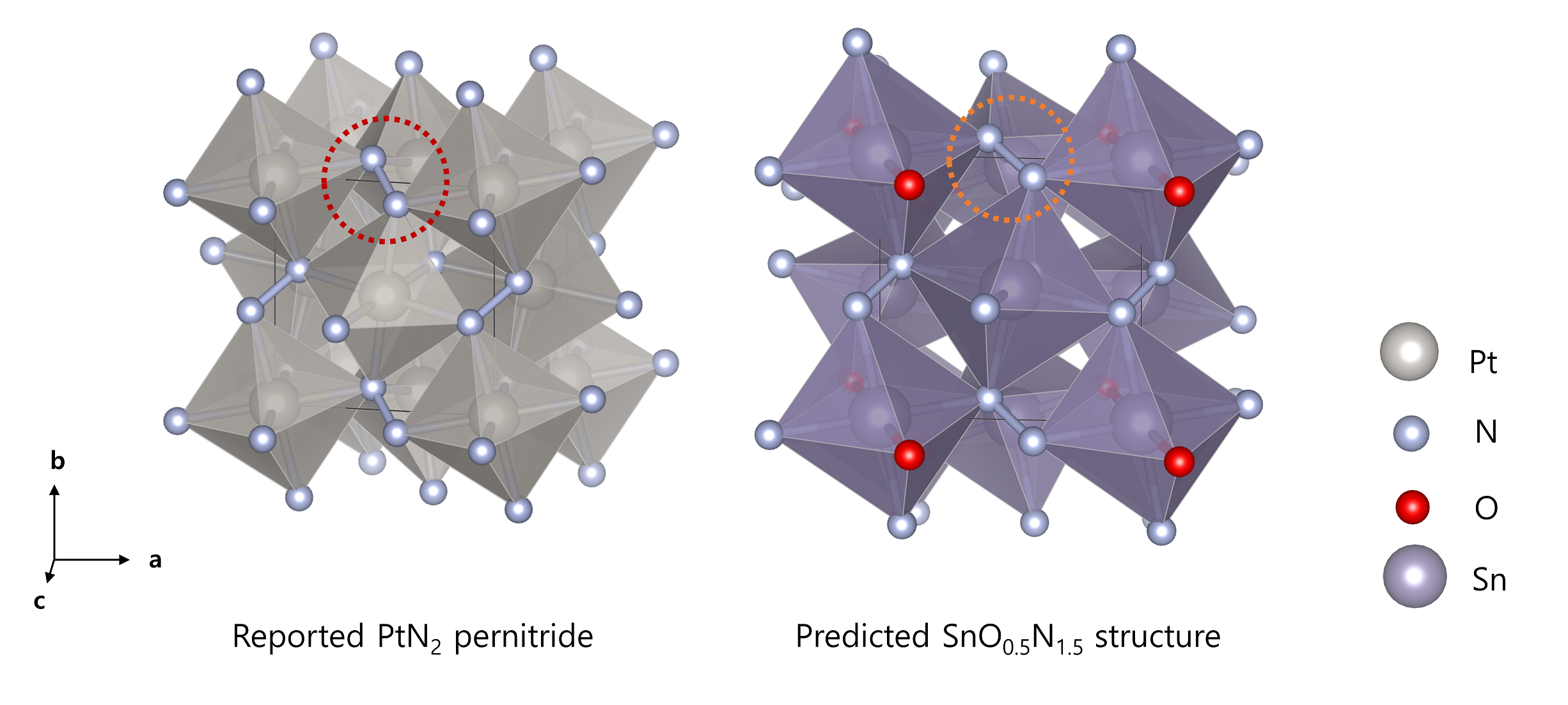


**Figure S3.** The reported PtN_2_ pernitride structure (left) and the predicted SnO_0.5_N_1.5_ structure (right). The two structures exhibit similar nitrogen dimer formation among the octahedra.


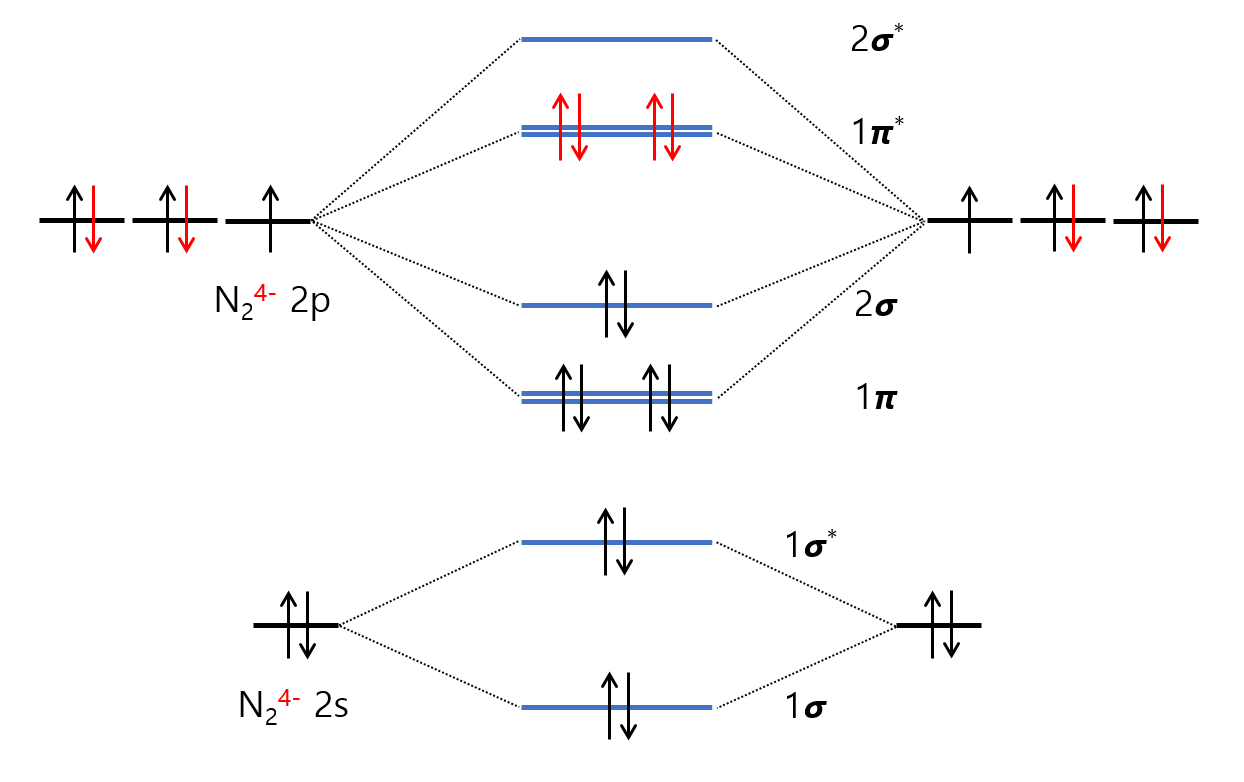


**Figure S4.** The molecular orbital diagrams for the nitrogen dimer. The neutral N_2_ dimer does not fill any antibonding states from 2p orbitals. When additional electrons are involved, states from 1𝞹* orbitals get filled in.


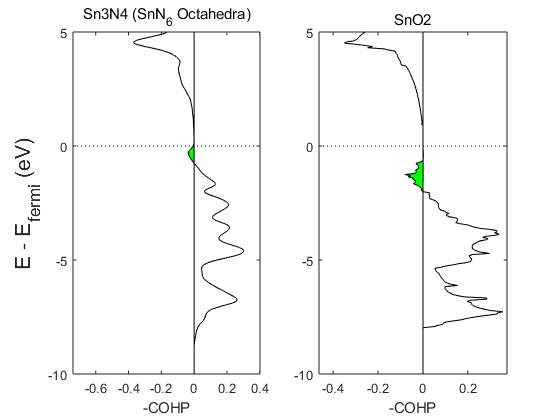


**Figure S5.** The COHP of Sn-N interaction from the SnN_6_ octahedra in Sn_3_N_4_ and Sn-O from SnO_2_. Both interactions involve filled antibonding states that are colored in green.


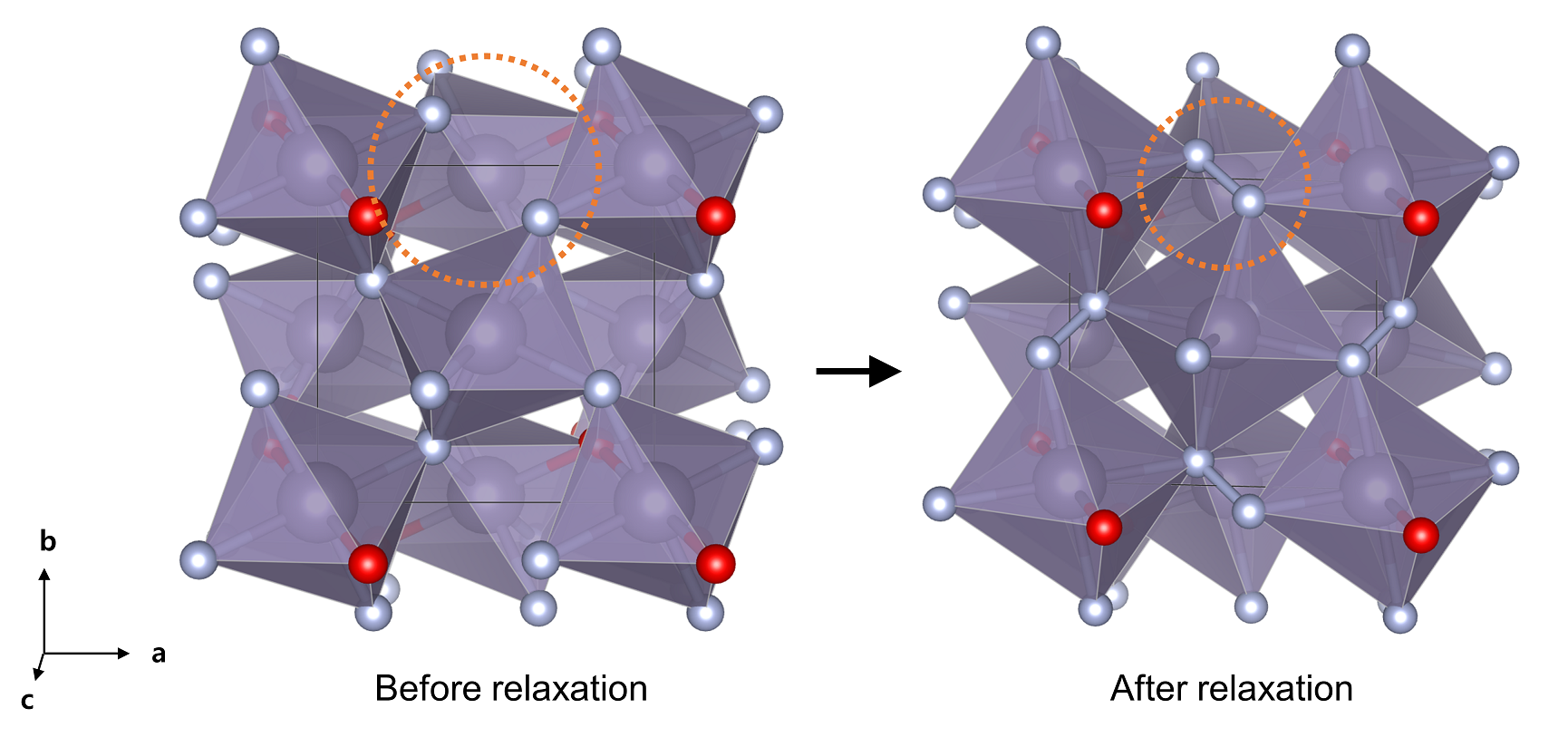


**Figure S6.** The configuration before atomistic relaxation (left) and after relaxation (right) by density functional theory energy minimization process. The relaxation process reveals collective polyhedral rotation that forms N-N bonds.


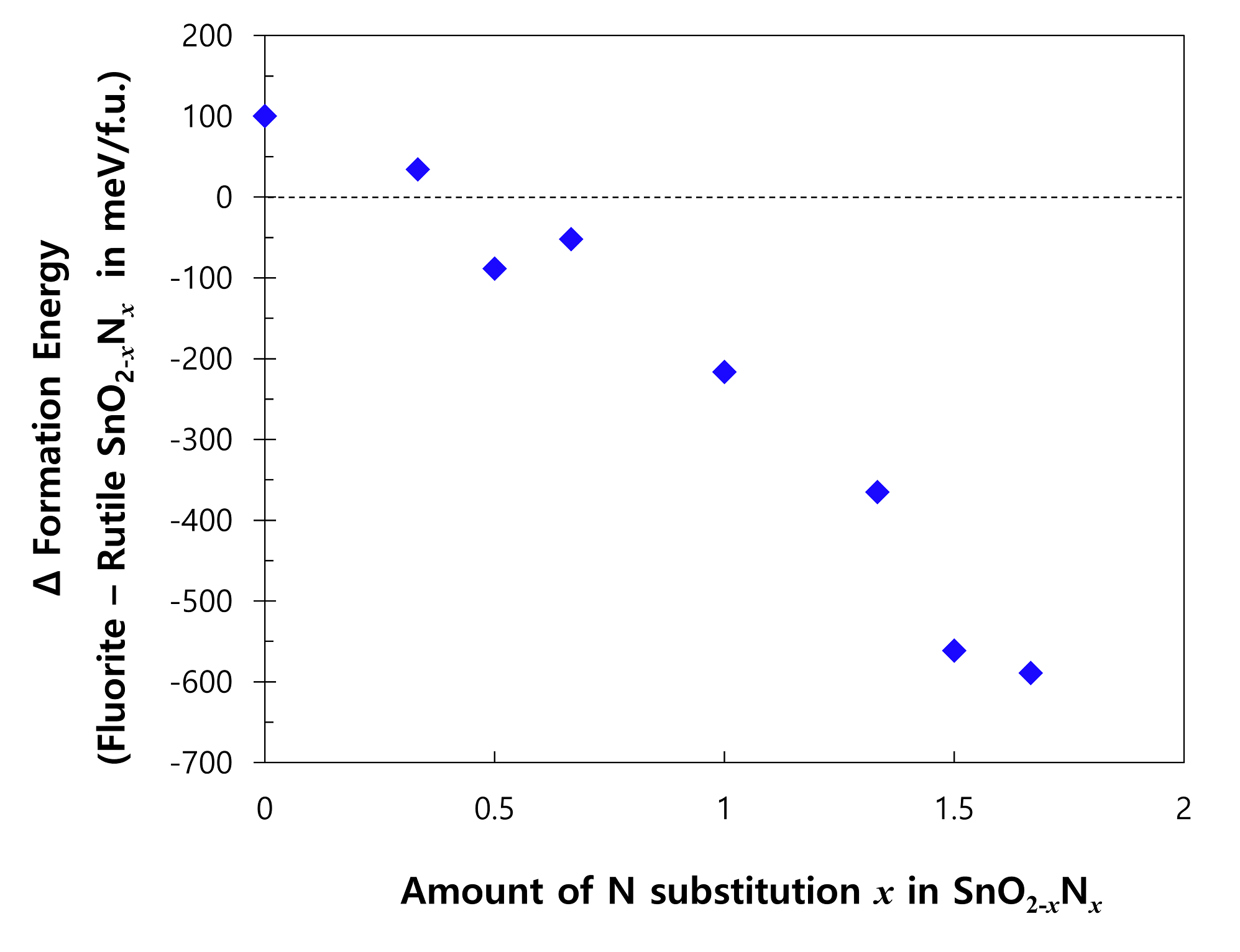


**Figure S7**. The difference in formation energy between fluorite and rutile phase SnO_2-_*_x_*N*_x_* with respect to the nitrogen substitution ratio *x*.

**Table S1.** Lattice constants for the lowest- and highest-energy SnO_0.5_N_1.5_ configurations in the fluorite structure.

| Configurations | a (Å) | b (Å) | c (Å) | α (°) | β (°) | γ (°) | # formula unit |
| --- | --- | --- | --- | --- | --- | --- | --- |
| Lowest-energy | 5.165 | 5.165 | 5.165 | 88.6 | 91.4 | 91.4 | 4 |
| Highest-energy | 5.362 | 5.208 | 10.859 | 89.9 | 90.0 | 90.0 | 8 |
